# Einfluss von Osteoporose auf physische Leistungsparameter von Personen im mittleren und höheren Lebensalter – eine Querschnittstudie

**DOI:** 10.1007/s00132-022-04329-3

**Published:** 2022-11-29

**Authors:** Guido Schröder, Dirk Flachsmeyer, Anne Bende, Julian Ramin Andresen, Reimer Andresen, Hans-Christof Schober

**Affiliations:** 1 Klinik für Orthopädie und Unfallchirurgie, Warnow Klinik Bützow, Am Forsthof 3, 18246 Bützow, Deutschland; 2 Medizinische Fakultät, Universität Rostock, Rostock, Deutschland; 3 Klinik für Unfall- und Wiederherstellungschirurgie, Charité Universitätsmedizin, Campus Benjamin Franklin, Berlin, Deutschland; 4 Institut für Diagnostische und Interventionelle Radiologie/Neuroradiologie, Westküstenklinikum Heide, Akademisches Lehrkrankenhaus der Universitäten Kiel, Lübeck und Hamburg, Heide, Deutschland; 5 Klinik für Innere Medizin IV, Klinikum Südstadt Rostock, Akademisches Lehrkrankenhaus der Universität Rostock, Rostock, Deutschland

**Keywords:** Knochenverlust, altersbedingter, Frakturen, spontane, Handgriffkraft, Betagte, Sarkopenie, Bone loss, age-related, Fractures, spontaneous, Hand grip strength, Oldest Old, Sarcopenia

## Abstract

**Hintergrund:**

PatientInnen im höheren Lebensalter, die an Osteoporose (OP) leiden, haben zusätzlich eine verminderte Muskelmasse und Muskelkraft – bekannt als Sarkopenie. Dies führt zu Funktionseinschränkungen sowie einem steigenden Sturz- und Verletzungsrisiko. Physische Leistungsparameter, wie Griff- und Rumpfkraft einerseits und die Gleichgewichtsfähigkeit andererseits, geben Auskunft über den neuromuskulären Allgemeinzustand und stellen einen Indikator der körperlichen Leistungsfähigkeit des alternden Menschen dar. Inwieweit stattgehabte osteoporotische Wirbelkörperfrakturen (VFs) zu einer Einschränkung der körperlichen Leistungsfähigkeit führen, wurde bisher nicht ausreichend untersucht.

**Material und Methoden:**

An der vorliegenden klinischen Untersuchung nahmen 118 Personen im Durchschnittsalter von 71,5 ± 9 Jahren teil. Es wurden zwei Gruppen gebildet – eine OP (58 PatientInnen) und eine Vergleichsgruppe (VG) (60 PatientInnen). In Subgruppenanalysen wurden OP-PatientInnen mit VFs und ohne VFs (0VFs) betrachtet. Für alle lag ein körperlicher Status mit Ergebnissen zu Handgriffkraft (HGS), Chair-Rising-Test (CRT), Tandemstand (TS), Tandemgang (TG) und Einbeinstand (EBS) vor. Alle erhobenen Daten wurden mit dem statistischen Softwarepaket SPSS, Version 23.0 analysiert.

**Ergebnisse:**

Zwischen den Gruppen OP und VG bestand hinsichtlich der Parameter HGS, CRT, TG, TS und EBS kein signifikanter Unterschied (*p* > 0,05). In der Subgruppenanalyse wiesen OP-PatientInnen mit VFs im Vergleich zu welchen mit 0 VFs eine geringere HGS auf (VFs: 24,3 ± 10,2 kg vs. 0 VFs: 29,7 ± 9,5 kg, *p* = 0,026). Der TS (VFs: 7,8 ± 3,2 s vs. 0 VFs: 9,5 ± 1,8 s, *p* = 0,008) wurde von OP-PatientInnen mit 0 VFs länger gehalten. Ihnen war es im TG möglich, mehr Schritte zu balancieren (VFs: 4,8 ± 3,0 vs. 0 VFs: 6,7 ± 2,4, *p* = 0,011). In einer Regressionsanalyse zeigten sich die Körpergröße, das Geschlecht und das Alter als unabhängige Einflussfaktoren auf die HGS (*p* < 0,001).

**Schlussfolgerung:**

Das PatientInnenalter, die Konstitution und das Geschlecht nehmen einen relevanten Einfluss auf die HGS, wobei die Ausgangsbedingungen nach diagnostizierter OP in dieser Altersgruppe auf vergleichbarem Niveau liegen. Bei einer Subgruppe von OP-PatientInnen mit VFs besteht ein enger Zusammenhang zwischen Knochen und Muskulatur mit einer zunehmenden Verschlechterung des muskuloskelettalen Systems. Zur Prophylaxe einer Osteosarkopenie erscheint ein frühzeitiges Training sinnvoll.

**Graphic abstract:**

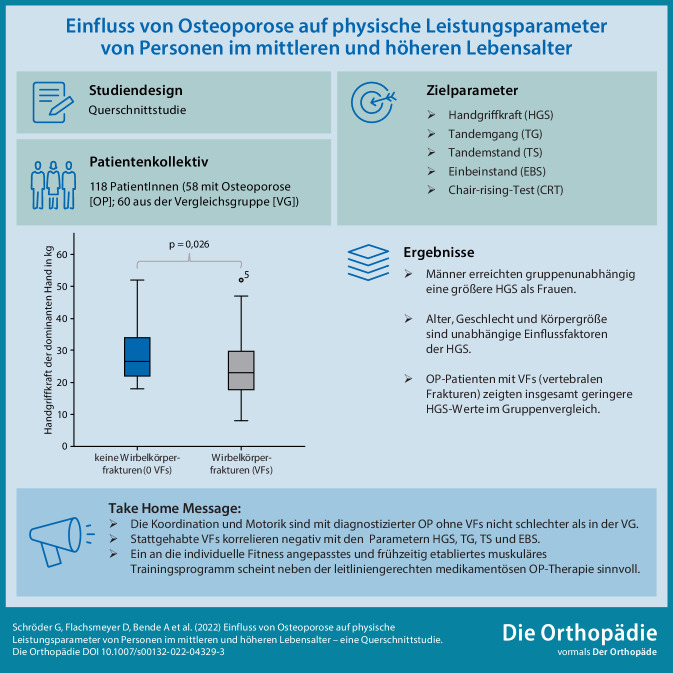

**Zusatzmaterial online:**

Die Online-Version dieses Beitrags (10.1007/s00132-022-04329-3) enthält eine weitere Tabelle. Die Tabelle listet die Grundcharakteristika der Studienpopulation auf.

## Abstract (en)

**Background:**

Osteoporosis (OP) in the elderly is accompanied by reduced muscle mass and reduced muscle strength, also known as sarcopenia. This results in functional limitations and a high risk of falls and injuries. The determination of physical performance parameters such as grip strength and trunk strength on the one hand, and balance abilities on the other, provide information about the individual’s general neuromuscular condition and serve as an indicator of physical performance in the elderly. The extent to which previous osteoporotic vertebral fractures (VFs) restrict an individual’s physical performance has not been adequately investigated yet.

**Material and methods:**

In total, 118 persons, with a mean age of 71.5 ± 9 years, participated in the clinical trial (ethics committee approval number A2020-0041). Patients were divided into an OP group (58 patients) and a control group (CG; 60 patients). OP patients with (VFs) and without vertebral fractures (0VFs) were viewed separately in the subgroup analysis. Data concerning physical status, including hand grip strength (HGS), the chair-rising test (CRT), tandem stance (TS), tandem gait (TG), and single-leg stance (SLS) were available for all patients. All data were analyzed using SPSS, Version 23.0.

**Results:**

No significant difference (*p* > 0.05) was registered between the OP and CG groups with regard of HGS, CRT, TG, TS, and SLS. In the subgroup analysis, OP patients with VFs had a lower HGS than OP patients without 0 VFx (VFs 24.3 ± 10.2 kg vs. 0 VFs 29.7 ± 9.5 kg, *p* = 0.026). TS was maintained longer by OP patients 0 VFs (VFs 7.8 ± 3.2 s vs. 0 VFs 9.5 ± 1.8 s, *p* = 0.008). The latter were also able to maintain their balance in TG over more numerous steps (VFs 4.8 ± 3.0 vs. 0 VFs 6.7 ± 2.4, *p* = 0.011). In a regression analysis, body size, gender, and age were shown to be independent factors influencing HGS (*p* < 0.001).

**Conclusion:**

Patient age, constitution, and gender have a relevant influence on HGS, with baseline conditions after diagnosed OP at comparable levels in this age group. In a subgroup of OP patients with VFs, there is a close relationship between bone and muscle with an increasing deterioration of the musculoskeletal system. For prophylaxis of osteosarcopenia, early training seems reasonable.

Mehr als die Hälfte aller Niedrigenergiefrakturen tritt bei Patienten ohne OP-typische Knochenmineralgehaltsänderung auf [50]. Klinische Untersuchungen im Hinblick auf die physische Fitness, insbesondere die Muskelkraft, können helfen, Frakturrisiken zu erkennen und Ansatzpunkte für eine Therapie sein. Die OP stellt für die betroffenen PatientInnen bei Auftreten von Frakturen (Fs) ein ernstes Problem dar. In Deutschland sind circa 8 Mio. Menschen von OP betroffen [22]. Die Inzidenz für klinisch auffällige osteoporotische VFs beträgt circa 1,4 Mio. weltweit [24].

## Einleitung

Ältere Menschen haben ein höheres Risiko für osteoporotische Frakturen, die zu einer schlechteren Lebensqualität, zu einer Behinderung, zum Verlust der Unabhängigkeit, zur Heimeinweisung und zu einer höheren Morbidität sowie Mortalität führen [27, 58]. Darüber hinaus entwickelt sich ein Verlust an konditionellen Ressourcen wie Muskelkraft, Ausdauer und Koordination, der die Aktivitäten des täglichen Lebens stark einschränkt [54]. Die Osteoporose (OP) steht in einem deutlichen pathophysiologischen Zusammenhang mit der Sarkopenie, einer altersbedingten Erkrankung, die mit einer Abnahme von Muskelmasse, -kraft oder -funktion einhergeht. Die Kombination dieser beiden Krankheiten wird als Osteosarkopenie bezeichnet [58]. Ein Verständnis der Pathophysiologie einer Osteosarkopenie ist neben den diagnostischen und therapeutischen Ansätzen der Schlüssel für eine optimale Sturz- und Frakturprävention bei älteren Erwachsenen [58].

Die Handgriffkraft (HGS) wird mit einer Vielzahl von Alterserscheinungen in Verbindung gebracht [9] und ist eine Schlüsselkomponente der Sarkopenie [11] sowie der Gebrechlichkeit [19]. Eine niedrige HGS erwies sich sogar als besserer Prädikator der Sterblichkeit als das Alter und der systolische Blutdruck [30, 34, 47]. Demnach besteht ein erhebliches Interesse an ihrer Rolle als Prädiktor für gesundes Altern und als potenzielles Instrument für die klinische Bewertung [10]. Es ist bereits bekannt, dass Faktoren, die ein gesundes Altern begünstigen, sowohl durch eine Erhöhung der im frühen Erwachsenenalter erreichten HGS als auch durch eine Abschwächung ihres späteren Rückgangs wirken können [55]. Es besteht daher auch ein Bedarf an normativen Daten für die HGS, die alle Phasen des Lebens – insbesondere die des alternden, osteoporotischen Menschen – abdecken.

Welchen Einfluss OP auf die motorische und die koordinative Entwicklung im Krankheitsverlauf hat, wurde bislang wenig untersucht. Insbesondere der durch OP-bedingte Wirbelkörperfrakturen (VFs) ausgelöste Schmerz ist für viele Betroffene von herausragender Bedeutung. Die Linderung dieser Schmerzen bei gleichzeitigem Erhalt der Muskulatur würde für PatientInnen einen subjektiven Therapieerfolg sowie einen Zugewinn an Lebensqualität darstellen. In der vorliegenden klinischen Untersuchung wurde dieser Frage nachgegangen.

Darüber hinaus sollten folgende Forschungsfragen mit der vorliegenden Studie beantwortet werden:

1. Gibt es einen Unterschied in der motorischen und koordinativen Leistungsfähigkeit von älteren PatientInnen mit und ohne OP?
2. Welche unabhängigen Faktoren beeinflussen die HGS?
3. Welche Zusammenhänge ergeben sich zwischen der HGS und anderen motorischen sowie koordinativen Fähigkeiten?

## Material und Methoden

### Studiendesign und Rekrutierung

Das Studiendesign entspricht einer retrospektiven und klinischen Untersuchung einer Behandlungsgruppe. Die Gruppenzuweisung erfolgte aufgrund einer nachgewiesenen OP in der Dual-Energy-X-ray-Absorptiometry-(DXA)-Messung. In der Subgruppenanalyse wurde OP-PatientInnen mit und ohne VFs betrachtet. Alle Teilnehmenden wurden umfassend über Methoden, Zwecke und Risiken des Studienprotokolls informiert. Sie erhielten zudem nach dieser Aufklärung eine schriftliche Erklärung zur Einwilligung bezüglich der Partizipation. Die Rekrutierung der Probandinnen und Probanden erfolgte einerseits über die ambulante Osteoporosesprechstunde des ortsansässigen Klinikums und andererseits über eine hausärztliche Praxis aus der Region.

### Ein- und Ausschlusskriterien

Einschlusskriterien der klinischen Untersuchung waren das Vorliegen einer nachgewiesenen therapiebedürftigen OP bei PatientInnen mit pathologischen Werten in der DXA (Lunar Prodigy, General Electric, Boston, MA, USA) sowie das Vorhandensein von Röntgenbildern der Brust- und der Lendenwirbelsäule.

In die Vergleichsgruppe (VG) wurden PatientInnen im Alter von mindestens 50 Jahren, mit einer alterstypischen Morbidität und dem Ziel, eine OP abklären zu lassen, eingeschlossen.

Zu Studienbeginn wurden für alle Partizipierenden eine körperliche Untersuchung im Sinne eines orthopädischen Status mit Handgriffkraft (HGS), Chair-Rising-Test (CRT), Tandemgang (TG), Tandemstand (TS), sowie Einbeinstand (EBS) durchgeführt.

Ausschlusskriterien waren alle Formen der schweren Herzinsuffizienz (NYHA-Klasse III und IV), ein schwer kontrollierbarer Hypertonus (persistierende Blutdruckwerte von ≥ 140/90 mm Hg trotz Einnahme von Antihypertensiva aus drei Medikamentenklassen) [40], relevante neuromuskuläre Defizite (u. a. Paresen, Hypästhesien, Apraxie, Adiadochokinese, Bewegungsstörungen, Tremor) eine Vestibulopathie und eine Betreuungspflichtigkeit.

### Klinische Tests

#### Handgriffkraft (HGS)

**Figure 1:**
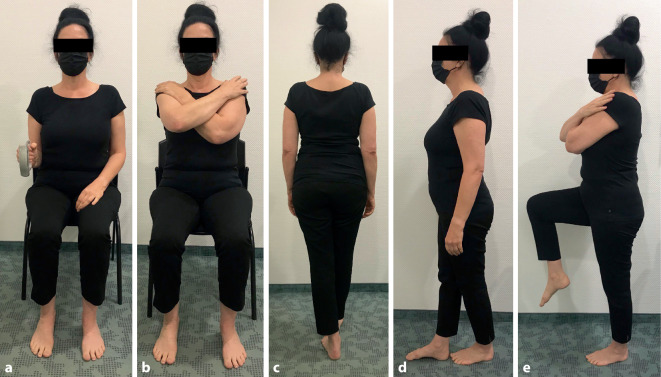

Die HGS in Kilogramm (kg) wurde mit dem Smedley-S-Dynamometer TMM Tokio 100 kg gemessen **(**Abb. 1a**)**. In Anlehnung an veröffentlichte Empfehlungen [44] nimmt die untersuchte Person zur Messung eine sitzende Position ein. Der Oberarm ist adduziert während der Ellenbogen um 90° gebeugt wird. Der Unterarm befindet sich, wie auch das Handgelenk in einer neutralen Position. Die Patientin oder der Patient führt zunächst mit der dominanten, dann mit der adominanten Hand je drei Versuche durch. Anschließend wird deren Mittelwert gebildet. Es ist erwähnenswert, dass Studien ähnliche Ergebnisse zeigen, unabhängig davon, ob in ihnen Durchschnitts- oder Maximalwerte verwendet wurden, die jeweils in mehreren Versuchen erzielt wurden [21].

Frühere Untersuchungen haben gezeigt, dass verschiedene Dynamometertypen und -marken ähnliche Ergebnisse liefern, das heißt, die Referenzwerte unabhängig vom verwendeten Dynamometertyp sind [15], und dass die mit einem Smedley-Dynamometer ermittelten Werte sehr stark mit denen korrelieren, die mit dem häufig verwendeten Jamar-Dynamometer erfasst wurden [57].

Bezüglich einer Sarkopenie gilt für Männer ein Wert < 27 kg als eingeschränkt leistungsfähig, wohingegen der Cut-off-Wert im Fall von Frauen bei 16 kg liegt [12]. Die HGS ist eine effiziente und einfache Methode, die Gesamtstärke bei älteren Menschen zu schätzen, und besitzt eine hohe sowie unabhängige Vorhersagekraft für funktionelle Einschränkungen und Behinderungen [43].

#### Chair-Rising-Test (CRT)

Der CRT in Sekunden (s) umfasst das fünfmalig nacheinander erfolgende Aufstehen und Hinsetzen von einem Stuhl ohne Armlehnen, wobei die Arme der Person vor der Brust verschränkt bleiben **(**Abb. 1b**)**. Die Ergebnisse geben Auskunft über ein normales oder erhöhtes Sturzrisiko – es gelten folgende Werte: Messung ≤ 10 s normal, Messung > 10 s erhöhtes Sturzrisiko [45]. Der Test wird ferner zur Beurteilung einer vorliegenden Sarkopenie durchgeführt [12].

#### Tandemgang (TG)

Beim TG soll die untersuchte Person acht Schritte auf einer Linie abschreiten **(**Abb. 1c**)**. Die Ergebnisse liefern zusätzliche Informationen über das Sturzrisiko. Sind acht Schritte möglich, ist das Ergebnis gleichbedeutend mit einem halbierten Sturzrisiko im Altersvergleich [45].

#### Tandemstand (TS)

Mit Hilfe des TS wird das Gleichgewicht bzw. die Koordination eines Menschen überprüft, wobei die Position zehn Sekunden lang zu halten ist **(**Abb. 1d**)**. Welcher Fuß dabei vorne steht, bleibt der Patientin/dem Patienten überlassen [45].

In Abb. 1 sind die einzelnen Tests exemplarisch dargestellt.

#### Einbeinstand (EBS)

Mit dem EBS wird ebenfalls die Koordination der PatientInnen getestet. Im vorliegenden Fall wurde der EBS mit offenen Augen auf dem dominanten **(**Abb. 1e**)**. Bein jeweils dreimal durchgeführt und anschließend wurde der MW gebildet. Die Probandinnen und Probanden wurden aufgefordert, die Arme, mit den Händen auf den Schultern, vor der Brust verschränkt zu halten. Das Knie des adominanten Beines wurde nach oben geführt, bis ein rechter Winkel zwischen dem Oberkörper und Oberschenkel erreicht wurde. Der Blick sollte im Testverlauf auf Augenhöhe und auf einen Gegenstand (z. B. auf der Wand) in fixer Position nach vorne gerichtet werden. Das Aufsetzen des Fußes führte zum Abbruch. Es existieren alters- und geschlechtsabhängige Normwerte: Für Frauen im Alter zwischen 70 und 79 Jahren liegt der Normwert bei 16,7 s, während er bei Männern dieser Altersgruppe bei 25,9 s erreicht [53].

### Schmerz

In der vorliegenden klinischen Untersuchung kam die Numerische Ratingskala (NRS) zur Anwendung. Es handelt sich dabei um eine eindimensionale, auf elf Einstufungsgraden basierende Schmerzskala, wobei der Wert null keinen und zehn den stärksten vorstellbaren Schmerz bedeutet. Innerhalb dieses Bereiches wählten die Probandinnen und Probanden die ihrem Schmerzempfinden entsprechende Zahl aus. Die Vorteile der NRS sind eine geringe Fehlerquote der Ergebnisse und eine zugleich hohe Akzeptanz bei Teilnehmenden [2].

### Knochendichtemessung

Die DXA ist das bei OP am häufigsten angewandte Vorgehen zur Knochendichtemessung. Sie wird von der Weltgesundheitsorganisation als Standardmethode zur messtechnischen Definition der OP erachtet [1].

Die Knochendichtemessung der Hüfte setzt sich aus vier Regionen zusammen: Femurhals, Trochanterregion, Intertrochanterregion, Ward’sches Dreieck. Am Achsenskelett werden hauptsächlich die Lendenwirbel erfasst. Der durch die Messung ermittelte T‑Score ergibt sich aus dem Vergleich der Dichtewerte der untersuchten Person mit denen eines durchschnittlichen jungen Erwachsenen. T‑Scores ≥ −1 entsprechen einem Normalbefund, T‑Scores −1 bis −2,5 einer Osteopenie und T‑Scores ≤ −2,5 einer Osteoporose [1]. Nach europäischen Leitlinien wurden für Männer und Frauen vorliegend auf Basis der DXA-Messung am Femurhals vier klinische Stadien (KS) definiert: KS 0 = Osteopenie, T‑Score zwischen −1 und −2,5 SD; KS 1 = T-Score < −2,5 SD; KS 2 = Manifeste OP, T‑Score < −2,5 SD, erste VF; KS 3 = fortgeschrittene OP, T‑Score < −2,5 SD, mehrere VFs und periphere Fs [1].

### Statistik

Die erhobenen Daten wurden mit dem statistischen Softwarepaket SPSS, Version 23.0 (SPSS Inc., Chicago, IL, USA) analysiert. Die quantitativen Merkmale sind als Mittelwert (MW), Standardabweichung (SD) und Anzahl der verfügbaren Beobachtungen (*n*) beschrieben sowie als Intervalls-MW ± SD dargestellt. Für die qualitativen Merkmale wird für die einzelnen Ausprägungen sowohl die absolute als auch die prozentuale Häufigkeit angegeben.

Für Gruppenvergleiche kam der Students t‑Test oder der Mann-Whitney-U-Test zum Einsatz. Die Auswahl erfolgte in Abhängigkeit vom Resultat des Shapiro-Wilk-Tests auf Normalverteilung. Darüber hinaus wurden Korrelationsanalysen in Abhängigkeit vom Skalenniveau durchgeführt. In einer multiplen Regressionsanalyse wurden unabhängige Einflussfaktoren auf die HGS ermittelt. Alle *p*-Werte sind das Resultat zweiseitiger statistischer Tests und prinzipiell wird *p* < 0,05 als statistisch signifikant angesehen.

## Ergebnisse

### Grundcharakteristika der Studienpopulation

An dieser klinischen Untersuchung nahmen 118 PatientInnen (91 w/27 m) teil. Das Alter der Partizipierenden lag zu Untersuchungsbeginn zwischen 51 und 93 Jahren (Gesamtdurchschnittsalter 71,5 ± 9 Jahre). Zwei Patientinnen und Patienten wurden aufgrund fehlender Dokumentation von der Studie ausgeschlossen. Unter den 58 OP-Erkrankten befanden sich 46 Frauen (79,3 %) und 12 Männer (20,7 %) im Durchschnittsalter von 71,7 ± 9,1 Jahren. Die 60 untersuchten Patientinnen und Patienten der VG umfassten 45 Frauen (75 %) und 15 Männer (25 %) im Durchschnittsalter von 71,3 ± 9,0 Jahren. Bezüglich der Alters- und Geschlechterverteilung ergaben sich zwischen den Gruppen keine signifikanten Unterschiede (*p* > 0,05). Der BMI war in der VG signifikant höher als in der OP-Gruppe (*p* < 0,001). Ein Anteil von 88,3 % der VG wies eine Fettleibigkeit auf. Im Vergleich dazu litten nur 63,8 % der OP-PatientInnen an einer Fettleibigkeit. Der Unterschied zwischen den Gruppen war diesbezüglich signifikant (*p* = 0,002). Dementsprechend befand sich in der VG ein signifikant höherer Anteil an Personen mit Diabetes mellitus (*p* = 0,002), arteriellem Hypertonus (*p* = 0,001) und koronarer Herzkrankheit (*p* = 0,013). Dagegen waren Partizipierende der Gruppe OP häufiger von einer Arthrose der oberen Extremität (*p* = 0,019) und der Wirbelsäule (*p* = 0,003) betroffen. Gleichzeitig lag mit 36,2 % vs. 8,3 % in dieser Gruppe ein höherer Anteil an PatientInnen mit Niereninsuffizienz vor (*p* < 0,001). Ein Gruppenvergleich hinsichtlich des Schmerzniveaus ergab einen signifikanten Unterschied zwischen OP und VG (*p* = 0,006). In der Subgruppenanalyse zeigte sich mit 45,8 % vs. 17,6 % in der OP-Gruppe mit VFs ein höherer Anteil an Osteoarthritis der Wirbelsäule (*p* = 0,020) und der oberen Extremität (62,5 % vs. 23,5 %, *p* = 0,003). Das Schmerzniveau lag bei OP-PatientInnen mit VFs (5 Punkte im Median) signifikant höher (*p* = 0,029) als bei OP-PatientInnen ohne VFs (1 Punkt im Median). Dementsprechend lag mit 29,2 % vs. 11,8 % in der Gruppe mit VFs ein höherer Anteil an PatientInnen mit regelmäßiger Analgetikaapplikation vor (*p* = 0,096). Das Ergebnis kann als statistischer Trend betrachtet werden. Der Anteil an regelmäßig Sportreibenden lag in der OP-Gruppe ohne VFs signifikant höher als in der Gruppe mit VFs (*p* = 0,023).

Die Tabelle im Zusatzmaterial online enthält die Grundcharakteristika der Studienpopulation. Der Knochenmineralgehalt (KMG) gemessen an der Lendenwirbelsäule lag bei den PatientInnen der OP-Gruppe ohne VFs im Durchschnitt bei −2,8 ± 0,6, bei den PatientInnen der OP-Gruppe mit VFs im Durchschnitt bei −3,4 ± 0,8. Die Unterschiede zwischen den Gruppen sind mit *p* = 0,001 signifikant.

### HGS

Die 118 Studienteilnehmenden konnten mit der dominanten Hand im Durchschnitt einen Wert von 28,3 ± 9,7 kg und mit der adominanten Hand von 24,8 ± 8,8 kg in der Handkraftmessung erzielen. Dabei wurden Werte zwischen 4 und 65 kg gemessen. Die Gruppe OP erreichte durchschnittlich mit der dominanten Hand eine HGS von 27,4 kg und mit der adominanten Hand von 24,1 kg. Diesbezüglich ergab sich kein signifikanter Unterschied zu den Durchschnittswerten der VG (*p* > 0,05, Tab. 1).

**Table 1:** 

Parameter	Gesamt (*n* = 118) MW ± SD	OP (*n* = 58) MW ± SD	VG (*n* = 60) M ± SD	*p*-Wert
HGS dominante Hand (kg)	28,3 ± 9,7	27,4 ± 10,1	29,1 ± 9,2	0,194
HGS adominante Hand (kg)	24,8 ± 8,8	24,1 ± 9,1	25,5 ± 8,5	0,288
CRT (s)	11,7 ± 5,1	11,3 ± 5,7	12,0 ± 4,6	0,133
TG (Schrittanzahl)	5,9 ± 2,5	5,9 ± 2,8	6,0 ± 2,2	0,606
TS (s)	9,1 ± 2,3	8,8 ± 2,6	9,3 ± 2,0	0,261
EBS (s)	8,2 ± 2,9	7,8 ± 3,4	8,6 ± 2,4	0,443

Daten sind als Mittelwert ± Standardabweichung (MW ± SD) dargestellt

*OP* Osteoporose, *VG* Vergleichsgruppe, *HGS* Handgriffkraft, *kg* Kilogramm, *CRT* Chair-Rising-Test, *s* Sekunde, *TG* Tandemgang, *TS* Tandemstand, *EBS* Einbeinstand

Für die VG wurden signifikante Korrelationen zwischen der HGS der dominanten und der adominanten Hand (r = 0,924, *p* < 0,001), der HGS und der Rumpfkraft (r = 0,302, *p* = 0,019), der HGS und dem TG (r = 0,387, *p* = 0,002) sowie der HGS und dem Lebensalter (r = −0,380, *p* = 0,003) erkannt.

Für die Gruppe OP zeigten sich signifikante Korrelationen zwischen der HGS der dominanten und dem entsprechenden Wert der adominanten Hand (r = 0,937, *p* < 0,001), der HGS und dem TG (r = 0,320, *p* = 0,014) sowie der HGS und dem Lebensalter (r = −0,560, *p* < 0,001). Zudem korrelierte die HGS der dominanten (r = 0,317, *p* = 0,015) und der adominanten Hand (r = 0,289, *p* = 0,028) in dieser Gruppe mit der aufgewandten Sportzeit. Die aufgewandte Sportzeit korrelierte wiederum mit dem T‑Score des linken (r = 0,386, *p* = 0,022) und rechten (r = 0,437, *p* = 0,018) Hüftgelenks sowie des rechten Schenkelhalses (r = 0,415, *p* = 0,044). Für den linken Schenkelhals lässt sich ein statistischer Trend erkennen (r = 0,353, *p* = 0,060).

**Figure 2:**
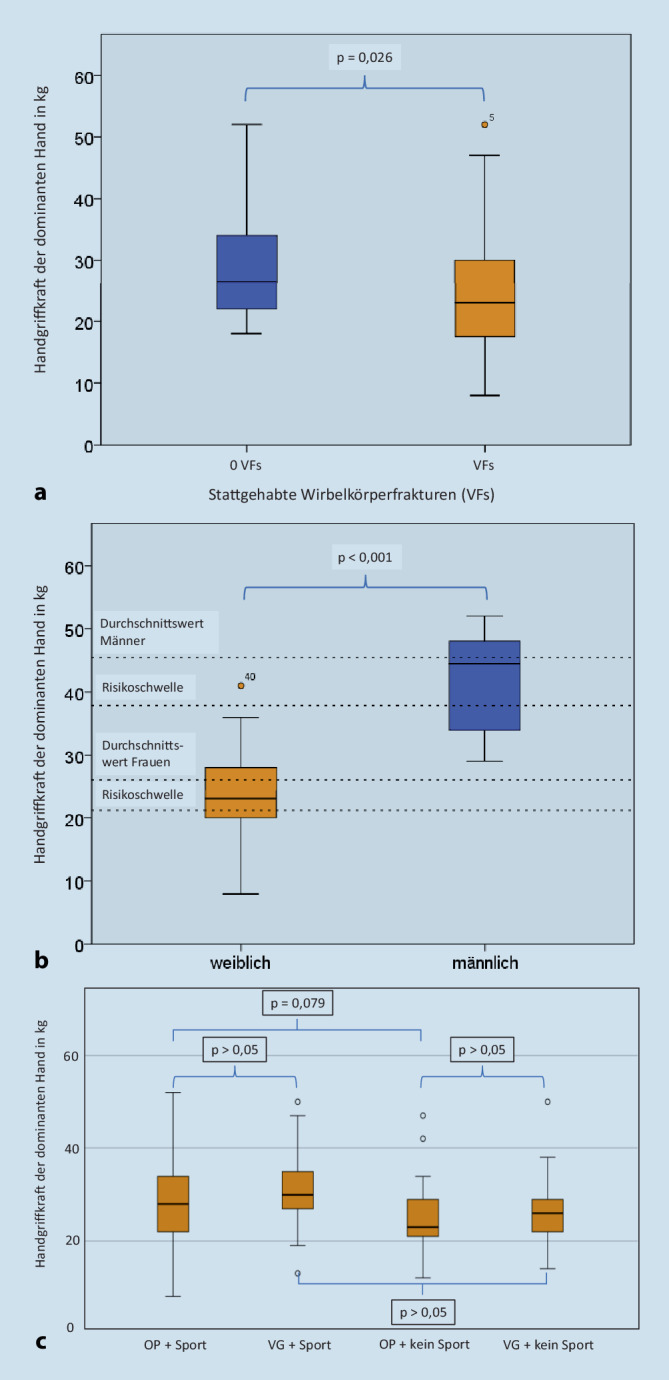

Bezüglich der Einteilung in OP mit- und ohne VFs zeigten sich signifikante Unterschiede in der HGS beider Hände. Die OP-PatientInnen mit VFs erreichten im Durchschnitt mit der dominanten Hand signifikant niedrigere Werte als welche ohne VFs (24,3 kg vs. 29,7 kg, *p* = 0,026, Abb. 2a). Frauen wiesen gruppenunabhängig eine niedrigere HGS auf als Männer (Tab. 2). Für die Gruppe OP ist der geschlechterabhängige Vergleich mit Referenzwerten und Risikoschwellen der Altersgruppen grafisch in Abb. 2b dargestellt. Regelmäßiges Sporttreiben ergab in der Gruppe OP einen statistischen Trend (*p* = 0,079, Abb. 2c). Gleichzeitig erzielte die Gruppe OP mit VFs niedere Werte mit der adominanten Hand (21,0 kg vs. 26,3 kg, *p* = 0,018). In Tab. 2 sind die HGS-Werte der Subgruppenanalyse zusammengefasst.

**Table 2:** 

Parameter	OP 0VFs (*n* = 34)	OP mit VFs (*n* = 24)	OP Frauen (*n* = 46)	OP Männer (*n* = 12)	VG Frauen (*n* = 45)	VG Männer (*n* = 15)	*p*-Wert ^a^	*p*-Wert ^b^	*p*-Wert ^c^	*p*-Wert ^d^	*p*-Wert ^e^
HGS dominante Hand (kg)	29,7 ± 9,5	24,3 ± 10,2	23,6 ± 6,3	42,2 ± 8,3	25,8 ± 5,7	39,0 ± 10,7	0,026	< 0,001	< 0,001	0,076	0,250
HGS-adominante Hand (kg)	26,3 ± 8,9	21,0 ± 8,6	20,8 ± 5,8	36,5 ± 9,0	22,9 ± 5,3	33,3 ± 11,3	0,018	< 0,001	0,001	0,118	0,231
CRT (s)	10,7 ± 4,7	12,3 ± 6,9	10,9 ± 4,1	13,1 ± 10,1	11,9 ± 4,1	12,1 ± 6,0	0,118	0,855	0,391	0,093	0,676
TG (Schritt-anzahl)	6,7 ± 2,4	4,8 ± 3,0	5,8 ± 2,8	6,1 ± 2,8	5,8 ± 2,3	6,5 ± 2,0	0,011	0,848	0,296	0,521	0,893
TS (s)	9,5 ± 1,8	7,8 ± 3,2	8,8 ± 2,6	8,5 ± 2,8	9,5 ± 1,6	8,9 ± 2,8	0,008	0,714	0,885	0,341	0,540
EBS (s)	8,4 ± 3,1	6,7 ± 3,6	7,9 ± 3,4	7,6 ± 3,3	8,7 ± 2,1	8,2 ± 3,0	0,050	0,717	0,807	0,543	0,611

Die Ergebnisse sind für sind für normalverteilte Parameter als Mittelwert ± Standardabweichung (MW ± SD) dargestellt

*OP* Osteoporose, *VFs* Wirbelkörperfrakturen, *0VFs* keine Wirbelkörperfrakturen, *HGS* Handgriffkraft, *CRT* Chair-Rising-Test, *TG* Tandemgang, *TS* Tandemstand, *EBS* Einbeinstand

^a^ OP 0VFs vs. OP VFs

^b^ OP Frauen vs. OP Männer

^c^ VG Frauen vs. VG Männer

^d^ OP Frauen vs. VG Frauen

^e^ OP Männer vs. VG Männer

### CRT

Die im CRT ermittelten Werte sind zwischen den Gruppen OP und VG nicht signifikant verschieden (*p* > 0,05). Ebenfalls nicht signifikant verschieden fiel der Vergleich der Werte von OP-PatientInnen mit sowie ohne VFs innerhalb der Subgruppenanalyse aus (*p* > 0,05).

### TG

Der Vergleich der Werte zwischen den Gruppen OP und VG ergab keinen signifikanten Unterschied (*p* > 0,05). Dagegen zeigte der Subgruppenvergleich, dass OP-PatientInnen ohne VFs signifikant mehr Schritte ausbalancieren können (*p* = 0,011).

### TS

Die Ergebnisse der Zeitmessung des TS ergaben zwischen den Gruppen OP und VG keinen signifikanten Unterschied (*p* > 0,05). Im Gegensatz dazu bestand ein signifikanter Unterschied zwischen OP-PatientInnen mit sowie ohne VFs (*p* = 0,008). Den OP-PatientInnen war es möglich, den TS länger zu halten.

### EBS

Im EBS wurde zwischen der Gruppe OP und VG kein signifikanter Unterschied erkannt (*p* > 0,05).

Dagegen zeigte sich ein statistischer Trend beim Vergleich der Werte von OP-PatientInnen mit sowie ohne VFs (*p* = 0,05).

### Multivariante Regressionsanalyse

In einer multivarianten Analyse wurden unabhängige Einflussfaktoren der HGS für die Gesamtgruppe bestimmt. Neben der Körpergröße (*p* < 0,001) bilden das Geschlecht (*p* < 0,001) und das Alter (*p* < 0,001) solche (Tab. 3).

**Table 3:** 

Einflussfaktoren	Standardfehler	Regressionskoeffizient	95 %-Konfidenzintervall untere, obere Grenze	Beta-Koeffizient	*p*-Wert
Alter	0,060	−0,300	−0,419, −0,182	−0,280	< 0,001
Geschlecht	1,622	8,679	5,467, 11,892	0,379	< 0,001
Körpergröße	0,079	0,420	0,264, 0,576	0,396	< 0,001

## Diskussion

Aus der vorliegenden Untersuchung folgt, dass PatientInnen mit der Diagnosestellung OP nicht zwangsläufig schlechtere muskuläre und koordinative Fähigkeiten aufweisen als gleichaltrige Vergleichspersonen (Tab. 1). Das ändert sich jedoch mit dem Auftreten von Frakturen, insbesondere von VFs (Tab. 2). Die physischen Leistungsparameter nehmen ab, während das Schmerzniveau deutlich steigt. Die OP-PatientInnen mit stattgehabten VFs wiesen eine signifikant niedrigere HGS sowohl der dominanten als auch der adominanten Hand auf. Dixon et al. [14] kamen in einer Untersuchung an 1265 Männern und 1380 Frauen im Alter von über 50 Jahren zum Schluss, dass eine niedrige HGS mit einem geringen KMG sowohl an der Wirbelsäule als auch an der Hüfte und einem erhöhten Risiko für eine VF verbunden ist. Diese Zusammenhänge können nach Angabe der genannten Forschenden durch Unterschiede in der Körpergröße oder im Lebensstil nicht erklärt werden. Auch Eguchi et al. [16] schlussfolgerten nach Abschluss ihrer Untersuchung an 1039 Frauen im Durchschnittsalter von 73 Jahren, dass der Verlust an Muskelmasse der unteren Extremität und der HGS eng mit dem Auftreten von VFs zusammenhängt. Die vorliegenden Ergebnisse unterstützen den Zusammenhang zwischen HGS und KMG jedoch nur indirekt. So korrelierte die HGS beider Hände mit der aufgewandten Sportzeit pro Woche. Letztere korreliert wiederum mit den gemessenen T‑Score-Werten der Hüftgelenke der OP-PatientInnen. Wie erwähnt gibt es in der Literatur viele Diskrepanzen in Bezug auf die Beziehungen zwischen Muskelkraft und Knochenmasse [4, 18]. Bei der Entstehung von Osteoporose spielen mehrere Faktoren eine Rolle, die teilweise für die unterschiedlichen Ergebnisse verantwortlich sein könnten. Neben hormonellen Faktoren wie einem Östrogenabfall [17, 28] ist erwiesen, dass viele andere Aspekte, darunter das Aktivitätsniveau, das Körpergewicht, die Körpergröße, der BMI, die Rauchgewohnheiten sowie die Alkohol- und die Kalziumzufuhr mit der Nahrung, ebenfalls einen Einfluss auf die maximale Knochenmasse bei Frauen haben [3, 32, 52, 56]. Den bei Frauen gut untersuchten, klaren Faktoren die zur OP führen, stehen wenige Aussagen zur OP-Entwicklung bei Männern gegenüber. Hier sind multiple Risikofaktoren, wie interkurrente Erkrankungen, Hypogonadismus, Medikamente und Lebensstilfaktoren relevant [51].

Eine Überprüfung aktueller Studien, die Referenzwerte für die HGS-Messung liefern, zeigt, dass die meisten von ihnen auf kleinen, nicht repräsentativen Stichproben beruhen. Nur vier frühere Studien enthalten landesweit repräsentative Referenzwerte für den gesamten Lebensverlauf – eine für den britischen Kontext [15], zwei für den US-amerikanischen Kontext [39, 42] und eine für den deutschen Kontext [55]. Diesen Forschungsarbeiten kann entnommen werden, dass bei Männern und Frauen die Spitzenmittelwerte der HGS im dritten und vierten Lebensjahrzehnt erreicht werden. In einer großen deutschen Studie wurden Referenzwerte für die HGS für Frauen und Männer unterschiedlicher Altersgruppen in Abhängigkeit von der Körpergröße veröffentlicht [55]. Neben der ethnischen Herkunft wurde ein markengleiches Dynamometer verwendet, das auch zur Einordnung der vorliegenden Daten dienen soll. Demnach erreichen Frauen im Alter zwischen 70 und 74 Jahren bei einer Körpergröße von 1,60–1,64 m eine mittlere HGS der dominanten Hand von 26,1 kg. In dieser Konstellation wird eine HGS von 21,2 kg als Risikoschwelle angesehen [55]. In der vorliegenden Studie verzeichneten Frauen der VG (71,4 Jahre, 1,61 m) im Mittel eine HGS der dominanten Hand von 25,8 kg. Sie lagen damit im Altersdurchschnitt auf vergleichbarem Niveau. Frauen mit OP (73,2 Jahre, 1,60 m) erzielten mit der dominanten Hand eine HGS im Mittel von 23,6 kg. Dieser Wert war der niedrigste im Gruppenvergleich, jedoch nicht signifikant verschieden zu den von Frauen der VG. Für Männer liegen die Referenzwerte der HGS bei 41,1 kg bei einer Risikoschwelle von 33,5 kg. Die Männer der VG (71,2 Jahre, 1,73 m) erreichten im Mittel mit der dominanten Hand eine HGS von 39,0 kg. Sie liegen damit etwas unter dem Altersdurchschnitt, aber deutlich über der Risikoschwelle. Für die Gruppe OP (66,2 Jahre, 1,77 m) liegt die Referenz der HGS der dominanten Hand bei 45,3 kg mit einer Risikoschwelle von 37,9 kg [55]. Auch hier wurde der Altersdurchschnitt mit 42,2 kg nicht erreicht. Die Männer mit OP weisen ebenfalls Ergebnisse deutlich über der Risikoschwelle auf. Die Cut-off-Werte für eine Sarkopenie wurden geschlechter- und gruppenabhängig nicht unterschritten.

Trotz methodischer Einschränkungen sollen zur Einordnung der vorliegenden Resultate weitere repräsentative Studien vorgestellt werden, die in der Regel keine radikal anderen Ergebnisse liefern. Basierend auf einer Zufallsstichprobe von 720 Teilnehmern konnten Peters et al. [41] zeigen, dass in den USA Spitzenmittelwerte von 49 kg für Männer und 29 kg für Frauen erreicht werden, wobei die Werte im siebten Lebensjahrzehnt auf 43 kg für Männer und 25 kg für Frauen abfallen. Diese Ergebnisse kommen denen von Perna et al. [39] bemerkenswert nahe, die auf der Grundlage der repräsentativen National Health and Nutrition Examination Study veröffentlicht wurden. In der vorliegenden Untersuchung gab es eine negative Korrelation zwischen der HGS und dem Lebensalter in beiden Gruppen. Mit Zunahme des Lebensalters sinkt die HGS signifikant, unabhängig davon, ob eine OP vorliegt. Neben dem Lebensalter waren auch die Körpergröße und das Geschlecht unabhängige Einflussfaktoren auf die HGS. Diese Ergebnisse stehen im Einklang mit den bereits vorgestellten Studien. Hervorzuheben gilt es, dass in beiden Gruppen eine Korrelation der HGS mit dem TG bestand. Der TG wird in der Geriatrie häufig zur Beurteilung der koordinativen Fähigkeiten im Rahmen einer Sturzrisikoevaluierung eingesetzt [46]. Das Ergebnis lässt vermuten, dass mit Zunahme der HGS der Rumpf leichter ausbalanciert werden kann, vergleichbare Studien liegen nach derzeitigem Kenntnisstand allerdings nicht vor. In der Subgruppenanalyse absolvierten OP-PatientInnen mit VFs weniger Schritte im TG. Zudem musste der TS früher abgebrochen werden. Dieses Resultat unterstützt die These, dass stattgehabte VFs zur Verschlechterung der koordinativen Fähigkeiten der OP-PatientInnen führen [33]. Hinsichtlich stattgehabter VFs und physikalischen Performanceparametern zeigt die Studie von Makarova et al. [33], dass VFs bei OP mit einer signifikanten Kraftabnahme aller Körpermuskeln einhergehen, insbesondere der tiefen Muskeln des Stabilisierungssystems der Wirbelsäule, d. h. des Rumpfstreckers (RE) und des Rumpfbeugers (RF). Zudem führen VFs zu einer ungünstigen Verteilung der Rückenmuskelkraft mit einem RE:RF-Verhältnis von 1:1 anstelle von 3:2, was sowohl bei Personen mit normaler als auch bei welchen mit unkomplizierter OP beobachtet wird. Bei PatientInnen mit VFs kam es ferner zu einer Verschlechterung der Stabilometrie und der Tests zur Bewertung der funktionellen Koordination, was nach Angaben der Forschenden auf Anomalien des statischen und dynamischen Haltungsgleichgewichts hinweist [33].

Das frühzeitige Erkennen von VFs ist demnach von Bedeutung, da sie ein deutlich erhöhtes Risiko für künftige osteoporotische Frakturen darstellen, die wiederum mit persistierenden Schmerzen, kyphotischer Deformation, Gewichtsverlust, Depression, verminderter Lebensqualität und sogar mit einer erhöhten Morbidität sowie Mortalität verbunden sind [29, 31, 36]. Die Inzidenz für klinisch auffällige osteoporotische VFs beträgt circa 1,4 Mio. weltweit [24]. Da die OP klinisch häufig stumm verläuft und die Diagnostik ausbleibt, wird sie erst spät oder gar nicht erkannt [25].

Die Rumpfmuskelkraft, gemessen mithilfe des CRT, korrelierte in keiner der Gruppen mit der HGS oder den T‑Score-Werten an der Lendenwirbelsäule. Bayramoğlu et al. [4] kamen in einer früheren Querschnittsstudie an 62 Frauen im Alter zwischen 41 und 76 Jahren zu vergleichbaren Resultaten. Die Forschenden schlussfolgerten, dass die isokinetische Kraft der Hüftabduktoren bei postmenopausalen Frauen mit und ohne Osteoporose nur schwach mit dem femoralen KMG korreliert. Die Rumpfmuskelkraft korrelierte in keiner der beiden Gruppen mit dem KMG der Lendenwirbelsäule. Die schwächere HGS, die sie bei den Frauen mit osteoporotischen Radien beobachteten, erklärten die Forschenden mit dem höheren Lebensalter der PatientInnen [4].

Wie eingangs erwähnt, gab es bereits zu Beginn einen signifikanten Unterschied in der Schmerzwahrnehmung sowohl der PatientInnen mit OP und VG als auch der mit und ohne VFs. Durch OP-bedingte Rückenschmerzen sind das Ergebnis von Frakturen und bzw. oder von mangelnder muskulärer Stabilisierung im Bereich der Columna vertebralis. Zur Behandlung der OP bestehen bereits eine Vielzahl therapeutischer Optionen medikamentöser [6, 8, 23, 35] und physiotherapeutischer Art [5, 7, 13, 20]. Regelmäßige körperlicher Bewegung führt unter anderem zur Linderung von Schmerzen, zur Verbesserung der Beweglichkeit und der Lebensqualität sowie zum Erhalt der Knochendichte [37, 38, 48, 49]. Gleichzeitig kann ein Beitrag zur Sturz- und Frakturprävention geleistet werden [26].

Als gemeinsamer Trend der vorgestellten Studien kann festgehalten werden, dass ein Training gelenknaher Muskeln zur Schmerzlinderung führt. Allerdings lässt sich dies aufgrund des abweichenden Untersuchungsgegenstandes nur begrenzt auf die vorliegende Studie übertragen. Besonders im Hinblick auf die Diagnose der PatientInnen ist weitere Forschung nötig.

## Limitationen

Das Querschnittdesign und die begrenzte Gruppengröße sind Limitationen der vorliegenden Studie. Komplexe statistische Verfahren konnten in ihrem Rahmen nicht angewandt werden, sodass sich die vorliegenden Ergebnisse nicht ohne Anpassungen verallgemeinern lassen. Darüber hinaus begrenzt die fehlende Verblindung sowohl der PatientInnen als auch der Untersuchenden die Aussagekraft der Studie. Für zukünftige Untersuchungen gilt es eine fortlaufende Evaluation bei längerfristiger Compliance der Teilnehmenden anzustreben, um die Evidenz zu verbessern.

## Fazit für die Praxis

- Die physikalischen Performanceparameter sind mit diagnostizierter Osteoporose (OP) ohne vertebrale Frakturen (VFs) nicht zwangsläufig schlechter als in einer gleichaltrigen Vergleichsgruppe.
- Das Alter, das Geschlecht und die Körpergröße sind unabhängige Einflussfaktoren der Handgriffkraft.
- Bei einer Subgruppe von PatientInnen mit OP sowie VFs besteht ein enger Zusammenhang zwischen Knochen und Muskulatur mit einer zunehmenden Verschlechterung des muskuloskelettalen Systems.
- Ein an die individuelle Fitness angepasstes und frühzeitig etabliertes muskuläres Trainingsprogramm scheint neben der leitliniengerechten medikamentösen OP-Therapie sinnvoll.

## Supplementary Information





## Abkürzungen

Abb.: Abbildung
BMI: Body-Mass-Index
CRT: Chair-Rising-Test
DXA: Dual-X-Ray-Absorptiometry
EBS: Einbeinstand
Fs: Frakturen
HGS: Hand Grip Strength
kg: Kilogramm
KMG: Knochenmineralgehalt
KS: Klinisches Stadium
LWS: Lendenwirbelsäule
MW: Mittelwert
N: Anzahl
NRS: Numerische Rating Skala
NYHA: New York Heart Association-Classification
OP: Osteoporose
RE: Rumpfextensor
RF: Rumpfflexor
s: Sekunde
SD: Standardabweichung
Tab.: Tabelle
TG: Tandemgang
TS: Tandemstand
u. a.: unter anderem
VG: Vergleichsgruppe
VF: Vertebrale Fraktur
VFs: Vertebrale Frakturen
