# Supplementary material for: Impact of osteoporosis on physical performance parameters of middle-aged and elderly individuals—a cross-sectional study
Source: Orthopadie (Heidelb). 2022 Nov 29;52(1):54–64. [Article in German] doi: 10.1007/s00132-022-04329-3 (PMC9842570; doi:10.1007/s00132-022-04329-3)
Supplement: Supplementary file 1 [file 132_2022_4329_MOESM1_ESM.pdf]

Tabelle: Grundcharakteristika der Studienpopulation

**Anamnese**	**OP Gesamt (n = 58)**	**OP 0VFs (n = 34)**	**OP VFs (n = 24)**	**VG Gesamt (n = 60)**	**p – Wert**[¥]	**p – Wert**[∞]
Alter (Jahre), MW ± SD	71,7 ± 9,1	71,0 ± 7,8	72,7 ± 10,7	71,3 ± 9,0	0,503[T]	0,815[T]
Geschlecht (männlich/weiblich)	12/46	7/27	19/5	15/45	0,982[C]	0,577[C]
Body-Mass-Index (kg/m^2), MW ±SD	26,7 ± 4,8	26,6 ± 4,4	26,8 ± 5,4	31,1 ± 5,4	0,880[T]	< 0,001[T]
Bone mineral density (T-Score LWS), MW ± SD	-3,1 ± 0,7	-2,8 ± 0,6	-3,4 ± 0,8		0,001[T]	
Schmerz (NRS 0 bis 10), Median (Q1 – Q3)	3 (0 – 6)	1 (0 – 5)	5 (1,25 – 6)	0 (0 – 4)	0,029[M]	0,006[M]
Regelmäßige Analgetika Substitution	11 (19 %)	4 (11,8 %)	7 (29,2 %)	7 (11,7 %)	0,096[C]	0,270[C]
Regelmäßiges Sporttreiben	32 (55,2 %)	23 (67,6 %)	9 (37,5 %)	29 (48,3 %)	0,023[C]	0,457[C]
Komorbiditäten						
Osteoarthritis obere Extremität	23 (39,7 %)	8 (23,5 %)	15 (62,5 %)	12 (20 %)	0,003[C]	0,019[C]
Osteoarthritis untere Extremität	32 (55,2 %)	21 (61,8 %)	11 (45,8 %)	25 (41,7 %)	0,230[C]	0,142[C]
Osteoarthritis Wirbelsäule	17 (29,3 %)	6 (17,6 %)	11 (45,8 %)	5 (8,3 %)	0,020[C]	0,003[C]
Rheumatoide Arthritis	13 (22,4 %)	8 (23,5 %)	5 (20,8 %)	9 (15,0 %)	0,808[C]	0,301[C]
Prolapsus nuclei pulposi	3 (5,2 %)	2 (5,9 %)	1 (4,2 %)	4 (6,7 %)	0,771[C]	0,731[C]
Arterieller Hypertonus	35 (60,3 %)	18 (52,9 %)	17 (70,8 %)	52 (86,7 %)	0,170[C]	0,001[C]

**Anamnese**	**OP Gesamt (n = 58)**	**OP 0VFs (n = 34)**	**OP VFs (n = 24)**	**VG Gesamt (n = 60)**	**p – Wert¥**	**p – Wert∞**
Diabetes mellitus	9 (15,5 %)	6 (17,6 %)	3 (12,5 %)	25 (41,7 %)	$0,594^{C}$	$0,002^{C}$
Hyperlipoproteinämie	20 (34,5 %)	12 (35,3 %)	8 (33,3 %)	26 (43,3 %)	$0,877^{C}$	$0,324^{C}$
Übergewicht	37 (63,8 %)	22 (64,7 %)	15 (62,5 %)	53 (88,3 %)	$0,863^{C}$	$0,002^{C}$
Hyperurikämie	3 (5,2 %)	3 (8,8 %)	0 (0,0 %)	4 (6,7 %)	$0,135^{C}$	$0,731^{C}$
Niereninsuffizienz	21 (36,2 %)	14 (41,2 %)	7 (29,2 %)	5 (8,3 %)	$0,349^{C}$	$< 0,001^{C}$
Myokardinfarkt	1 (1,7 %)	1 (2,9 %)	0 (0,0 %)	4 (6,7 %)	$0,397^{C}$	$0,183^{C}$
Apoplex	1 (1,7 %)	0 (0,0 %)	1 (4,2 %)	1 (1,7 %)	$0,230^{C}$	$0,981^{C}$
Herzinsuffizienz (NYHA-Klasse I und II)	4 (6,9 %)	2 (5,9 %)	2 (8,3 %)	5 (8,3 %)	$0,717^{C}$	$0,769^{C}$
Koronare Herzkrankheit	4 (5,9 %)	2 (5,9 %)	2 (8,3 %)	14 (23,3 %)	0,717	$0,013^{C}$
Herzklappenerkrankung	2 (3,4 %)	1 (2,9 %)	1 (4,2 %)	7 (11,7 %)	$0,801^{C}$	$0,093^{C}$
Hypothyreose	9 (15,5 %)	4 (11,8 %)	5 (20,8 %)	17 (28,3 %)	$0,347^{C}$	$0,093^{C}$
Hyperthyreose	4 (6,9 %)	1 (2,9 %)	3 (12,5 %)	1 (1,7 %)	$0,157^{C}$	$0,159^{C}$
Asthma bronchiale	2 (3,4 %)	2 (5,9 %)	0 (0,0 %)	2 (3,3 %)	$0,227^{C}$	$0,972^{C}$
Chronisch obstruktive Lungenerkrankung	5 (8,6 %)	1 (2,9 %)	4 (16,7 %)	6 (10,0 %)	$0,067^{C}$	$0,797^{C}$
Periphere arterielle Verschlusskrankheit	3 (5,2 %)	1 (2,9 %)	2 (8,3 %)	4 (6,7 %)	$0,361^{C}$	$0,731^{C}$
Chronisch venöse Insuffizienz	1 (1,7 %)	0 (0,0 %)	1 (4,2 %)	5 (8,3 %)	$0,230^{C}$	$0,102^{C}$
Hepatitiden	3 (5,2 %)	0 (0,0 %)	3 (12,5 %)	4 (6,7 %)	$0,034^{C}$	$0,731^{C}$
Alkohol Abusus	0 (0,0 %)			3 (5,0 %)		$0,085^{C}$

**Anamnese**	**OP Gesamt (n = 58)**	**OP 0VFs (n = 34)**	**OP VFs (n = 24)**	**VG Gesamt (n = 60)**	**p – Wert¥**	**p – Wert∞**
Chronisch inflamma-torische intestinale Erkrankung	1 (1,7 %)	1 (2,9 %)	0 (0,0 %)	0 (0,0 %)	$0{,}397^{C}$	$0{,}307^{C}$
Gastroösophageale Refluxkrankheit	3 (5,2 %)	1 (2,9 %)	2 (8,3 %)	5 (8,3 %)	$0{,}361^{C}$	$0{,}495^{C}$
Malignom Erkrankung	3 (5,2 %)	1 (2,9 %)	2 (8,3 %)	2 (3,3 %)	$0{,}361^{C}$	$0{,}620^{C}$
Depression	7 (12,1 %)	4 (11,8 %)	3 (12,5 %)	9 (15,0 %)	$0{,}933^{C}$	$0{,}642^{C}$
Epilepsie	0 (0,0 %)			1 (1,7 %)		$0{,}323^{C}$
Restless Legs Syndrom	2 (3,4 %)	1 (2,9 %)	1 (4,2 %)	3 (5,0 %)	$0{,}801^{C}$	$0{,}676^{C}$

Die Ergebnisse sind für nicht normalverteilte Parameter als Median mit Angabe des 1. und 3. Quartils (Q1–Q3) sowie für normalverteilte Parameter als Mittelwert ± Standardabweichung (MW ± SD) dargestellt. Chi-Quadrat-Test^{C}, Students-t-Test^{T}, Mann-Whitney-U-Test^{M}, ¥, Vergleich OP 0VFs vs. OP VFs; ∞, Vergleich OP vs. VG.

Abkürzungen: OP, Osteoporose; VG, Vergleichsgruppe; LWS, Lendenwirbelsäule; NRS, Numerische Rating Skala; NYHA, New York Heart Association-Classification
